# Chronic Heat Stress Part 2: Increased Stress and Fear Responses in F_1_ Pekin Ducks Raised from Parents That Experienced Heat Stress

**DOI:** 10.3390/ani13111748

**Published:** 2023-05-25

**Authors:** Esther Mary Oluwagbenga, Victoria Tetel, Jenna Schober, Gregory S. Fraley

**Affiliations:** Animal Sciences, Purdue University, West Lafayette, IN 47907, USA; eoluwagb@purdue.edu (E.M.O.); vtetel@purdue.edu (V.T.); jschober@purdue.edu (J.S.)

**Keywords:** parental hormone, phenotype plasticity, offspring performance, HPA response, behavior

## Abstract

**Simple Summary:**

There are many factors that can cause stress in poultry, but one of the most significant is high temperatures in the environment, which can make production difficult. It is important to assess the impact of heat stress (HS) on breeder ducks and their offspring (F_1_ generation) to identify biomarkers that can measure the response of poultry to HS effectively. This approach can reveal both physical and genetic characteristics that can serve as useful markers to determine the susceptibility or tolerance of different poultry breeds to HS. The results of our study indicated that exposing the parents to HS resulted in heightened Hypothalamic–Pituitary–Adrenal (HPA) axis and fear responses in their offspring. We concluded that the maternal hormones that are deposited into eggs during HS may influence the early programming of the brain, leading to alterations in the HPA and behavioral response in the F_1_ generation.

**Abstract:**

The effects of HS on the welfare of poultry have been reported to have a transgenerational effect on phenotype plasticity. The goal of our experiment was to determine whether parental exposure to HS would impair the performance, HPA axis response, or behavior of their offspring. We treated adult drakes and hens (n = 80 ducks/treatment) at peak lay with HS or the control temperature for 3 weeks and incubated eggs collected from the last 3 days of the experiment. We utilized 76 ducklings/parental treatment group: control (CON-F_1_) and HS (HS-F_1_). Weekly data for body weights, body condition scores (BCSs), and novel object test (NOT) were collected. At 3 weeks of age, the ducks (n = 6/treatment) were subjected to adrenocorticotropic hormone (ACTH/cosyntropin, 0.0625 mg/kg) challenge or vehicle as the control. Blood samples were collected at 0, 1, 2, 3, and 4 h relative to treatment for serum glucocorticoid and heterophil-to-lymphocyte ratio (HLR) analyses. All injected birds were euthanized with pentobarbital on the second day relative to ACTH administration, and the spleen and bursa were removed and weighed immediately. Duck level analyses were completed using one- or two-way ANOVA as appropriate. BCSs were analyzed using a chi-squared test. The HS-F_1_ ducks had a lower hatch weight (*p* < 0.05) compared with the CON-F_1_ ducks but no significant difference in growth rates during the 5-week period. NOT (n = 4) analyses showed that the HS-F_1_ ducks had a greater fear response (*p* < 0.001) compared with the CON-F_1_ ducks. Similarly, an ACTH stimulation test showed that the HS-F_1_ ducks had significantly (*p* < 0.05) heightened corticosterone and HLR responses compared with the CON-F_1_ ducks. The HS-F_1_ ducks showed altered baseline and ACTH-stimulated levels of cortisol compared with the controls. Our data suggest that parental exposure to HS impacts the HPA response and fearfulness of the F_1_ generation in Pekin ducks.

## 1. Introduction

Glucocorticoid (GC)—primarily cortisol and corticosterone—secretion from adrenal cortical cells is controlled by adrenocorticotropic hormone (ACTH) secreted from the anterior pituitary. In birds, ACTH secretion by the anterior pituitary is primarily controlled by corticotropin-releasing hormone (CRH) and arginine vasotocin (AVT) secreted from the median eminence of the hypothalamus [1,2,3,4]. Plasma concentrations of GC start to rise within minutes of being exposed to a stressor, with the amount of secretion dependent upon the duration and intensity of the stressor; this physiological response is used to restore homeostasis following a stressor. Several studies have reported an increase in circulating levels of both cortisol and corticosterone following exposure to different types of stressors in domestic ducks [5,6,7,8], broiler chickens [9,10,11], Japanese quail [12,13,14], layers [15,16], and zebra finches [17]. Recent studies have suggested that cortisol, but not corticosterone, is selectively deposited into the egg albumen [18,19]. However, it is not known if the deposition of this hormone into eggs is related to changes in offspring phenotypic plasticity, a condition referred to as the maternal effect. 

The maternal effect refers to the ability of a mother to influence the characteristics of her offspring through the transfer of nutrients or hormones such as testosterone, androstenedione, progesterone, pregnenolone, estradiol, norepinephrine, or GC into the egg (reviewed by [20]). Avian eggs contain hormones that can transmit information from one generation to the next, and this demonstrates how the hen can influence the phenotypic plasticity of her offspring [21]. Many studies have focused on the effects of gonadal hormones on offspring phenotype, but other hormones that play a role in maintaining homeostasis, such as GC, are also likely to have a significant impact [22]. The transfer of GC from the mother to the egg, possibly through the yolk or albumen, can have a profound impact on the phenotypic characteristics of the developing offspring [19,23]. Several studies have reported the effect of in ovo administration of corticosterone on the embryonic and post-hatch development of the offspring [24,25,26]. Some of these effects include reduced hatchability, lower hatching and post-hatch body weight, reduced competitive ability, lower post-hatch growth rate, increased embryo mortality, and higher fearfulness [27,28]. Hayward et al. [23] reported a decrease in hatching weight and growth rate and an increase in HPA axis response to restraint stress in the offspring of Japanese quail that were implanted with corticosterone. However, we currently do not know how chronic heat stress, similar to that experienced by poultry due to climate change, impacts the physiology and behavior of the offspring of domesticated ducks. 

In a previous study by our lab [18], adult Pekin duck breeders were given cyclical heat stress for 3 weeks. We demonstrated that prolonged heat stress led to a reduction in welfare and increase in physiological indicators of stress in a sex-dependent manner; hens, but not drakes, showed increases in corticosterone, cortisol, and heterophil-to-lymphocyte ratios [18]. Further, we showed that heat stress resulted in a significant reduction in egg quality parameters and a reduction in egg production in those hens [18]. We also observed an increase in cortisol, but not corticosterone, levels in the egg albumen following 3 weeks of heat stress [18]. Incubation of eggs collected from the last 3 days of the heat stress or control periods showed a reduction in the percentage of fertilized eggs and in hatchability [18]. The purpose of our current study was to evaluate the effects of maternal exposure to heat stress (HS) on the offspring. We evaluated the F_1_ generation from the previous study [18] in terms of their growth, physical characteristics of welfare, fearfulness, and GC response to an ACTH challenge. Our results suggest that maternal exposure to heat stress causes lower hatch body weight but does not impact future growth or body weight gain. Further, maternal heat stress is related to greater glucocorticoid release and fear responses to ACTH and novel object test (NOT), respectively.

## 2. Materials and Methods

### 2.1. Animals

HS and control eggs were placed in the same incubator (NatureForm Model 2340, Integrated Hatcheries, Jacksonville, FL, USA). A total of 270 eggs were collected from the last 3 days of the breeder heat stress (or control, [18]) condition and incubated as per industry standards [29]. Given the reduced hatchability of the HS parents’ eggs [18], the control hatchlings were randomly culled so that the two flocks had equal numbers. The F_1_ generation was brooded (beginning with 31.5 °C and reducing to 22 °C by 2 weeks of age) and raised as grow-out meat ducks, following published standards, under an 18:6 (L:D) light cycle [29]. Ducks were placed into floor pens and standard density (0.28–0.85 m^2^; brood and grow-out, respectively) at each age of the grow-out period [29] with pine litter shavings covering 2/3 of the floor. Ducks were given ad lib access to water via nipple lines (8 ducks/nipple, 40 mL/min approximate flow rate) with mesh flooring over a pit underlying the nipple lines. Ducks were fed industry-standard starter diet until 10 days of age, then placed on standard grower diet for the remainder of the experiment. All protocols were approved by the Purdue Animal Care and Use Committee (PACUC # 2109002195).

### 2.2. Experimental Design

Upon hatching, all ducklings’ hatch body weights were obtained to calculate means (and standard error of the means as reported below). The number of ducks in the CON-F_1_ flock was reduced to equal that of the HS-F_1_, and each flock was housed in different single pens in the same room to minimize any environmental or management variables. Each flock was placed in a single pen as opposed to multiple pens of small numbers due to the increase in hierarchical and aggressive behaviors associated with ducks housed in small numbers [29,30,31,32,33]. We utilized 76 ducks per treatment pen. The ducks were exposed to an 18:6 light cycle (white LED, 3500 K, ~1.6 × 10^3^ μM photons/m^2^/s at the level of the ducks’ heads).

### 2.3. Production and Welfare Assessment

All ducks were weighed on weeks 0, 1, 2, 3, 4, and 5, with 0 representing the day of hatch. Body condition scores (BCSs) such as of foot pad quality, eyes, nostrils, and feather cleanliness and quality were also assessed for all ducks (n = 76/treatment) at weeks 0, 1, 2, 3, 4, and 5 (Table 1). A higher BCS indicates a decline in welfare [34,35,36].

### 2.4. ACTH Challenge

An ACTH challenge was given at 3 weeks of age, during midweek between days for NOT and BW assessments. For the ACTH challenge, ducks were given intra-muscular injections of cosyntropin, a synthetic form of ACTH (amino acids 1–24; 0.0625 mg/kg). Saline with 0.1% mannitol (ACTH vehicle) was used as the vehicle control. Randomly selected, apparently healthy ducks were injected with either ACTH or vehicle for controls (n = 6/treatment). Blood samples (n = 6/treatment/pen) were collected from the tibial vein of the same ducks into tubes at 0, 1, 2, 3, 4, and 5 h relative to the administration of ACTH or vehicle for the determination of serum GCs and to produce blood smears for heterophil-to-lymphocyte ratios (HLRs). Blood samples were centrifuged, and the serum was collected and stored at −20 °C until assayed by ELISA for GCs. Complete blood counts were performed on the blood smear by certified pathologists (Antech Diagnostics, Chicago, IL, USA) unaware of the experimental treatments. The heterophil count was divided by the lymphocyte count to obtain the HLR. All injected birds were euthanized with pentobarbital on the second day relative to ACTH administration, and the spleen and bursa were removed and weighed immediately. Organ weights are expressed relative to body weight (g/kg).

### 2.5. ELISA for Glucocorticoids

The kits utilized for this project were from Cayman Chemicals (corticosterone: kit #16063; cortisol: kit #560360), and the assays were run according to the manufacturer’s recommendations. Details of the kit verification have been reported previously [7,37]. Plates were incubated with samples overnight at 4 °C. For the development of the plate, Ellman’s Reagent was reconstituted with 50 mL of ultrapure water. A total of 200 μL of this reagent was added to each well on the plate before being placed on an orbital shaker for 90 min. At 90 min, plates were read at 405 nm (SynergyLx, Biotek; Santa Clara, CA, USA).

### 2.6. Novel Object Test (NOT)

NOT was performed beginning at 7 days of age (week 1) and again at weeks 2, 3, and 4 to obtain behavioral responses from ducks in the same pen. NOT was completed on the same day as body weight measurements but was performed before any researcher or farm staff entered the rooms. NOT procedure was performed according to a previous report with modifications [38]. We used a red laser light as the novel object. An individual controlling the laser stood about 3.5 m outside the pen, and the laser point placed at a fixed spot was then slowly moved around the pen. WYZE Cam V3 cameras (Kirkland, WA, USA) were placed in the pens, one for each pen, and recorded continuously during the test. Each camera was able to visualize its entire respective pen. Videos were assessed by scan sampling every ten seconds across the full minute, and behaviors were recorded using JWatcher software along with Excel. Percentage of ducks performing each analyzed behavior was averaged across the 6 bins of time for each test. Responses of ducks within the same pen were categorized as either “fearful” (moving or running away from the light), “passive” (looking at the light or holding still), and “approach” (moving towards the light). All the tests were carried out by the same person and analyzed by a single individual. The videos were analyzed by a single individual unaware of the treatment groups by pausing the video every 10 s and counting the number of ducks showing each response. Ducks were counted only within 1 duck’s length of the laser dot in order not to count movements not directly associated with the novel object. The 1 min time frame of the test was selected so that ducks would not habituate to the procedure, and 1 min was sufficient to evaluate all ducks in a given pen.

### 2.7. Statistical Analyses

The duck was considered the statistical unit. Hormone and HLR data were analyzed by a 2-way repeated measures ANOVA (Linear Mixed Model, treatment x time using compounded symmetry for 2 parameters) with time as the repeated measure (MacJMP, SAS Institute, Cary, NC, USA). Post hoc analyses were performed by Tukey–Kramer pairwise comparison test. The NOTs were replicated 4 times over the grow-out period, thus giving an n = 4 per parental treatment. The overall analyses of the NOT between treatment groups was performed by a student’s *t*-test for each behavioral state. Body condition scores were analyzed using a chi-squared test. A *p* ≤ 0.05 was considered significant.

## 3. Results

### 3.1. Production and Welfare Assessment

**Figure 1:**
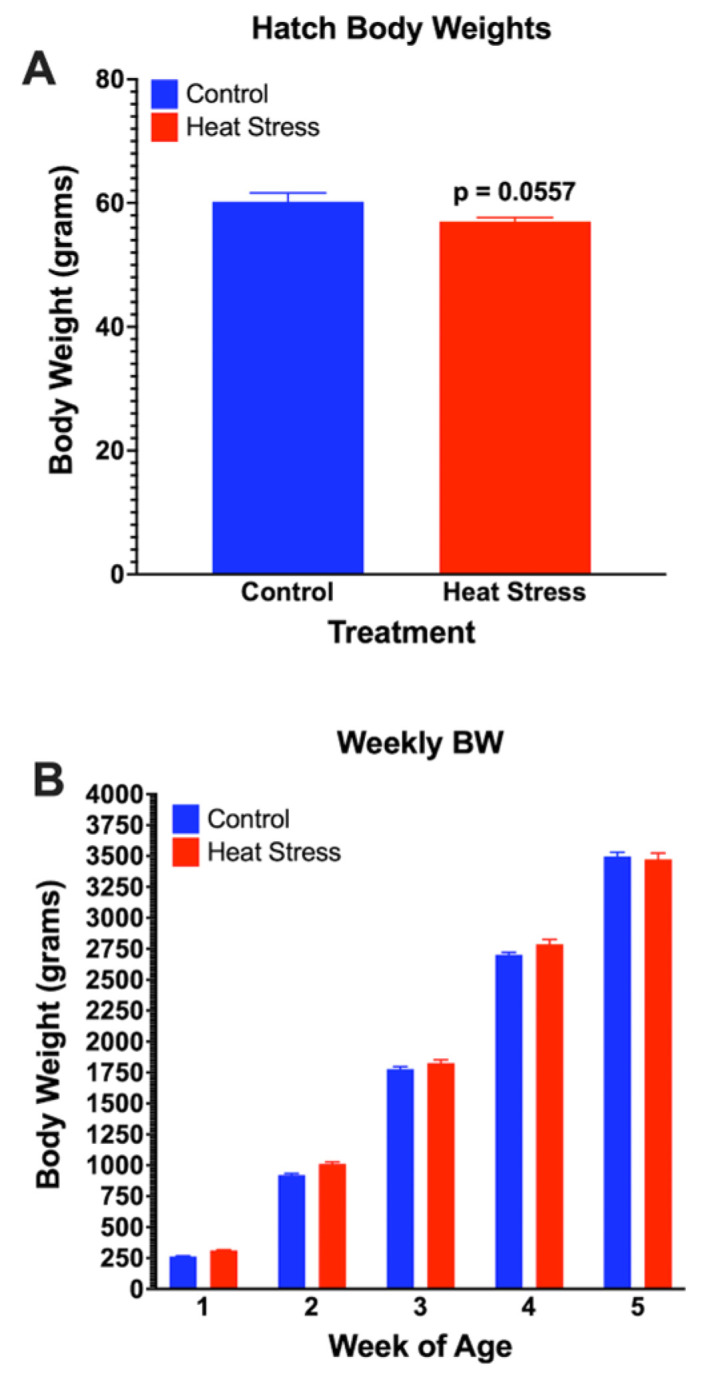
Hatch weight (**A**) and body weight (**B**) of ducks from parents exposed to cyclic HS or control. Data shown are means ± SEM, n = 76/treatment.

**Figure 2:**
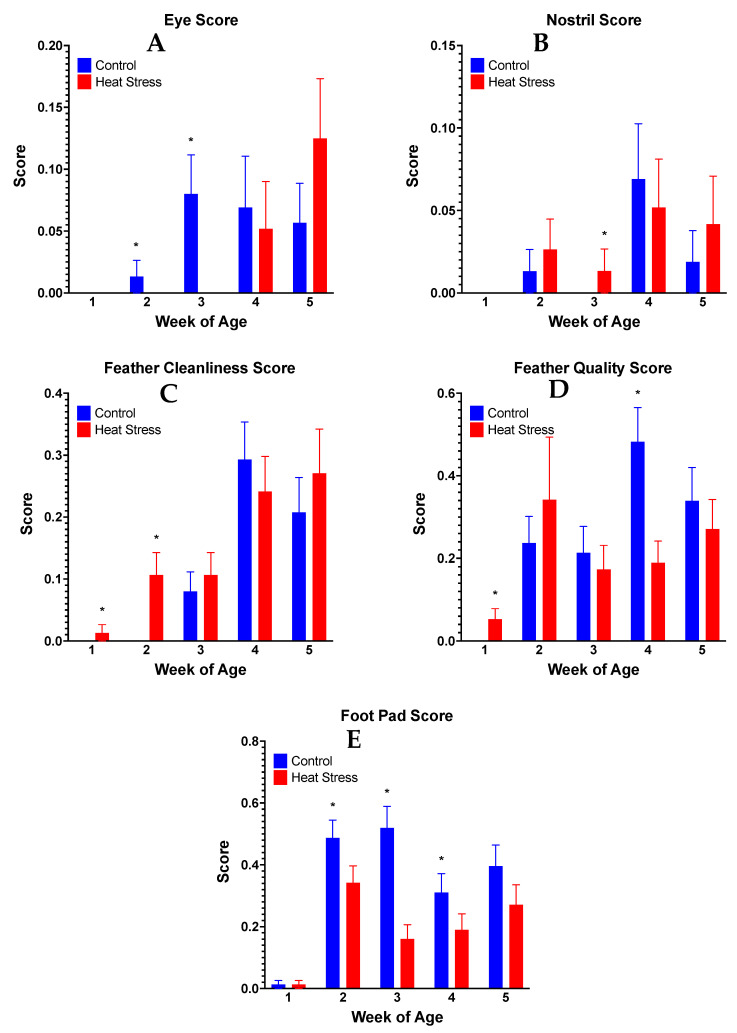
Welfare assessment score for eye quality (**A**), nostril quality (**B**), feather cleanliness (**C**), feather quality (**D**), and foot pad quality (**E**) in ducks from parents exposed to cyclic HS or control. Data shown are means ± SEM, n = 76/treatment/week. * *p* < 0.05.

There was no mortality in either of the two flocks. The HS-F_1_ ducks had a (*p* = 0.0557) lower hatch weight compared with the CON-F_1_ ducks (Figure 1A). However, the body weights during the 5-week grow-out period were similar between the HS and CON-F_1_ ducks (Figure 1B). The eye quality score increased significantly (*p* = 0.0488) in the CON-F_1_ ducks at weeks 2 and 3 (Figure 2A), and the nostril score increased significantly (*p* = 0.042) in the HS-F_1_ ducks at week 3 (Figure 2B). In addition, the feather cleanliness score increased significantly (*p* = 0.0036) in the HS-F_1_ ducks at weeks 1 and 2 (Figure 2C), and the feather quality score increased significantly (*p* = 0.0431) in the CON-F_1_ ducks at week 4 (Figure 2D). Finally, the foot pad quality score increased significantly (*p* = 0.0256) in the CON-F_1_ group at weeks 2, 3, and 4 (Figure 2E). Although a higher BCS indicates a decline in welfare, all scores were well below 1.0, thus indicating very good overall body conditions in both groups.

### 3.2. ACTH Challenge

**Figure 3:**
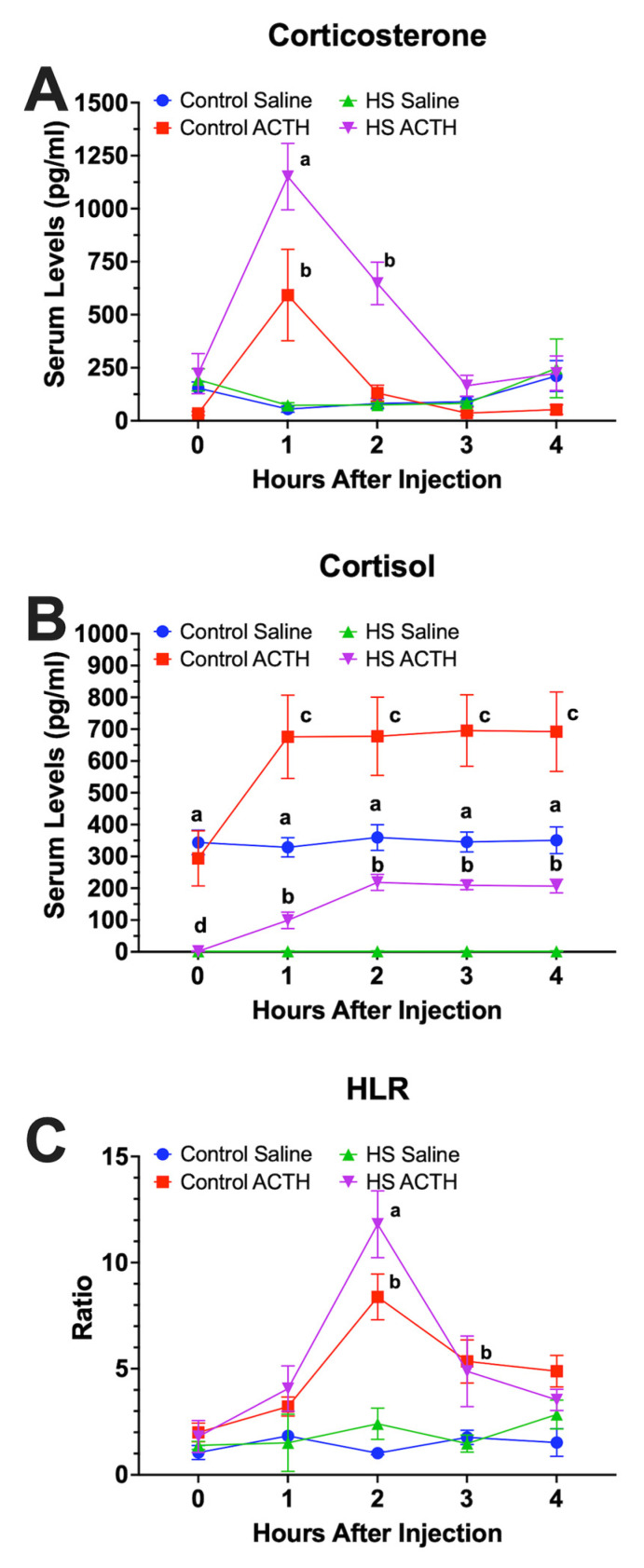
Serum corticosterone (**A**), cortisol levels (**B**), and heterophil-to-lymphocyte ratio (**C**) in ducks from parents exposed to cyclic HS or control following ACTH administration. Data shown are means ± SEM, n = 6/treatment/hour. Letters indicate statistically different groups, *p* < 0.05.

The HS-F_1_ group showed a heightened corticosterone response to ACTH compared with the CON-F_1_ group. Circulating levels of corticosterone were significantly (*p* < 0.0078) elevated 1 and 2 h after ACTH in the HS-F_1_ ACTH-treated group compared with all other groups. Circulating levels of corticosterone increased significantly (*p* < 0.0013) in the CON-F_1_ group only 1 h after ACTH injection compared with the vehicle-treated controls (Figure 3A). Baseline levels of cortisol in the HS-F_1_ group were just above detectable limits and significantly (*p* < 0.001) lower than baseline levels in the CON-F_1_ group at all time points. Circulating levels of cortisol increased significantly (*p* = 0.0085) at hours 1, 2, 3, and 4 in the CON-F_1_ and HS-F_1_ groups following ACTH compared with the vehicle-injected ducks in each group. However, the ACTH-stimulated levels of cortisol were significantly higher in the CON-F_1_ compared with HS-F_1_ group (Figure 3B).

**Figure 4:**
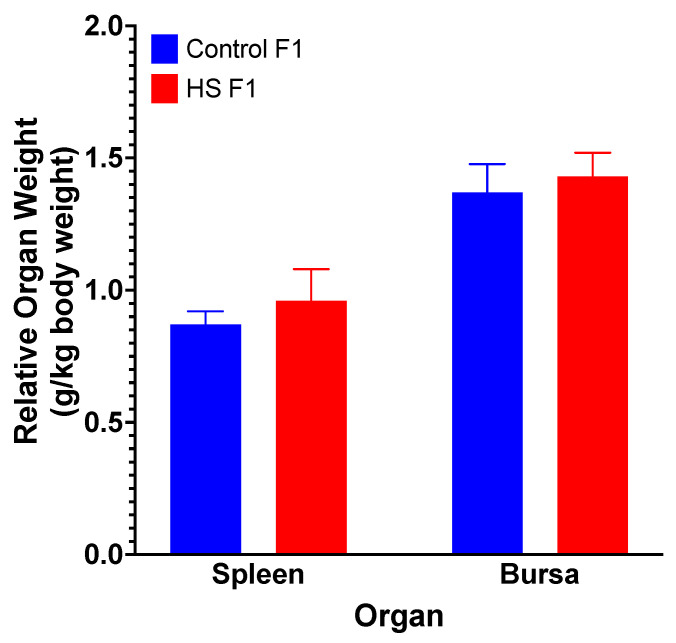
Relative weights of spleen and bursa in ducks from parents exposed to cyclic HS or control following ACTH administration. Data shown are means ± SEM, n = 6/treatment.

HLR was significantly (*p* < 0.004) higher at 2 h in the HS-F_1_ ACTH-treated group than in all other groups (Figure 3C). ACTH-treated ducks in both the HS-F_1_ and CON-F_1_ groups showed significantly (overall, *p* < 0.001) greater HLR levels then the vehicle-treated controls from both groups. No significant (*p* > 0.05) differences in the relative weights of the spleen or bursa of Fabricius (Figure 4) were observed between the HS-F_1_ and CON-F_1_ groups.

### 3.3. Novel Object Test

**Figure 5:**
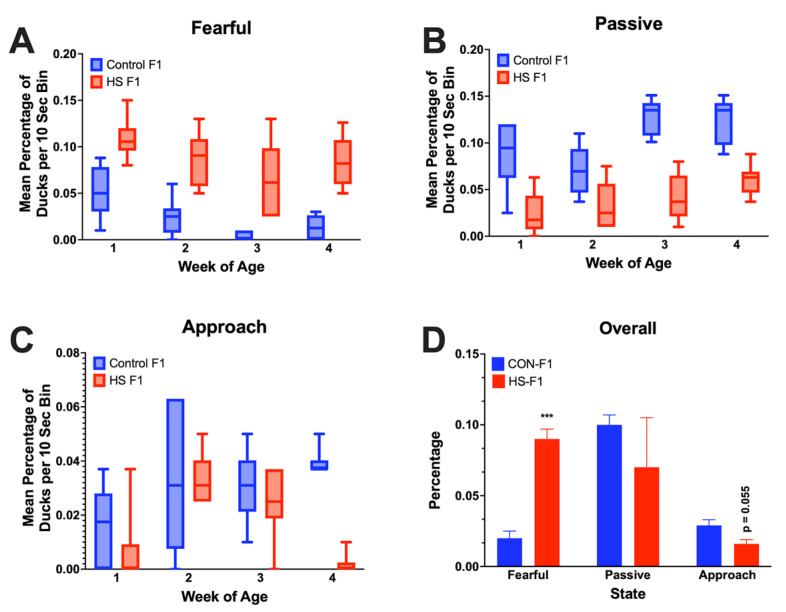
Novel object test responses for fearful (**A**), passive (**B**), and approach (**C**) in ducks from parents exposed to cyclic HS or control over 4 weeks of production. Panel (**D**) illustrates overall statistical analyses from all 4 weeks of NOTs (n = 4 per parental treatment). *** *p* < 0.001.

Weekly NOTs showed that the HS-F_1_ group had a greater percentage of ducks that elicited a fear response at all time points and a lower number of ducks that showed a passive response at all time points. Overall, NOT analyses revealed that the HS-F_1_ group showed a significantly increased (*p* < 0.001) fear and decreased passive response compared with the controls and a reduction in the percentage of ducks who approached the novel object (*p* = 0.055). Figure 5 illustrates the NOT data.

## 4. Discussion

The purpose of our study was to determine whether parental exposure to heat stress would impair the post-hatch performance, ACTH response, welfare, or behavior of their offspring. To achieve these goals, we exposed adult breeder ducks to a 3-week heat stress or other periods as controls [18]. The F_1_ offspring were then evaluated for stress-related phenotypes. We observed that HS-F_1_ ducks had a lower hatch weight compared with CON-F_1_ ducks; however, the growth rates during the 5-week grow-out period were not significantly different between the two flocks. Repeated NOTs showed that the HS-F_1_ ducks had a greater fear response compared with the CON-F_1_ ducks. Similarly, an ACTH stimulation test to drive GC release showed that the HS-F_1_ ducks had significantly (*p* < 0.05) heightened corticosterone and HLR responses compared with the CON-F_1_ ducks. The HS-F_1_ ducks also showed altered baseline and ACTH-stimulated levels of cortisol compared with the controls. Our data suggest that parental exposure to HS has an impact on the phenotype of the F_1_ generation.

The increase in egg GC levels that we observed from the parental ducks [18] could alternatively indicate maternal conditions in terms of age, welfare, environmental condition, and genetics that are transferred as cues to the offspring. This information transfer can alter the phenotype of offspring, thereby affecting both pre-hatch and post-hatch welfare and development [21,39]. Our study showed that the exposure of duck hens to chronic HS significantly lowered the hatching weight of their offspring but did not elicit significant differences in the growth rate or weekly body weights between the ducklings from the HS and control hens. These results support those from other studies that demonstrated that parental stress impairs embryo development and hatchability [40]. Parental HS can affect embryo development, hatchability, or chick performance through hormonal disorder and poor sperm penetration and morphology [41,42]. High levels of corticosterone in the hen’s blood can affect reproductive hormone concentrations in the egg as GCs are considered to have antigonadotrophic properties that affect the availability of nutrients for the embryo [40,43,44]. Although some reports suggest that chronic HS in parents elicits poor results in offspring, our data may suggest otherwise.

Body condition scoring (BCS) is an evaluation system used by many as an indirect measure of welfare in commercial poultry, including ducks, and it involves a visual assessment of the ducks to assign quality scores for their eyes, nostrils, feathers, and foot pads [18,35,36,45,46]. A previous study in our lab reported that HS significantly increased the BCSs of breeder ducks compared with controls, suggesting a decline in welfare [18]. However, there is a dearth of research on transgenerational effects, namely, how parents’ BCSs may relate to the BCSs of their offspring. Some of our data may have an apparent large standard error; however, the range of the Y axes suggests that is not the case. The data from the current study for welfare scores, GC and HLR levels, and body weights are comparable to previous studies in ducks [7,18,34,35,36,37]. Bowers et al. [47] reported that the offspring of wild songbirds that were fed corticosterone-injected worms showed a superior body condition before fledging (fledging refers to the stage between birth and the growth of feathers that enable flight or independent action) compared with the controls. Overall, the lack of negative effects on the BCS and growth rate in our HS-F_1_ group in this study suggests that maternal GCs do not negatively impact offspring welfare, at least as presented by changes in BCS. However, it is possible that the results would change if the birds were exposed to environmental conditions similar to those experienced by the parents.

Hormones serve as a link between genotype and phenotype that is used to interpret environmental cues to produce phenotype variability that influences physiology, behavior, and welfare [48]. One of the ways of mitigating the effect of stress is by enhancing the HPA response of animals to stress, thereby improving their coping mechanism. Exposure to maternal GC may be beneficial as it is important for the early programming of the brain and behavior [49]. Welberg and Seckl [50] reported that an increase in anxiety may lead to increased cautiousness, thus improving survival skills in an adverse environment in mammals. In a comprehensive study, quail offspring whose parents were exposed to chronic HS had a higher inflammatory response than quail offspring whose parents were not exposed to chronic heat stress [51]. De Fraipont et al. [52] observed that the offspring of stressed female lizards showed higher risk evasion than those of control females. Previous studies used corticosterone in eggs to study the effect of maternal hormones on embryo and offspring development; however, recent studies strongly support that cortisol, not corticosterone, is deposited in eggs [18,19,53,54]. Heiblum et al. [55] compared the effect of in ovo administration of cortisol and corticosterone and reported that both GCs resulted in embryo mortality while cortisol led to a decrease in the hatch weight and body weight of white leghorn chickens. Bowers et al. [47] reported that offspring of wild songbirds fed corticosterone-injected worms showed a higher begging rate for food compared with controls. Yet another study reported increased flight performance of juvenile European starlings following in ovo exposure to corticosterone [56]. However, these parental effects are not limited to birds according to a study showing that female mammals exposed to elevated levels of GC during pregnancy may pass on adverse effects to their offspring, including feminization, anxiety behaviors, and a hypersensitive HPA axis [57,58,59,60]. These previous studies support our current results that showed increased HPA and fear responses in the offspring of heat-stressed hens following the ACTH stimulation and NOT, respectively. Our results further suggest that maternal GC may participate in the early programing of the brain to alter HPA activity and behaviors in response to stress.

There are limited resources on the effect of maternal GC on immune parameters in poultry. However, some studies reported that maternal hormones in the egg enhanced the immune response of offspring in chickens [61,62,63,64], ducks [65], penguins [66], great tits [67], and yellow-legged gulls [68]. Videla et al. [51] reported that quail offspring whose parents were exposed to chronic HS had a higher HLR response than quail offspring whose parents were not exposed to chronic HS. Although we found no significant differences in the weights of the spleen and bursa in the F_1_ ducks, the HS-F_1_ ducks did show a heightened HLR response compared with the CON-F_1_ ducks, which supports the previous findings that the deposition of maternal hormones into the egg may enhance the immune response of the offspring. However, further research is required to determine whether this heightened HLR response translates to an enhanced response to an immunological challenge. Future studies need to confirm the fear response with additional behavioral analyses: are the increased GC, HLR, and fear responses of F_1_ ducks from heat-stressed parents ultimately good or bad for our poultry production?

## 5. Conclusions

In conclusion, these data, combined with our previously published findings on heat stress in adult breeder ducks [18], show that maternal exposure to chronic heat stress may exaggerate the HPA response and fear behavior of their offspring. The transfer of environmental cues to the egg is a means of priming the offspring for adverse environmental conditions similar to that of the hen, as evident in the greater HPA and fear response for adaptability, and cortisol may be an important player in transferring this information.

## Figures and Tables

**Table 1:** Body condition scoring rubric for welfare assessment ^1^.

	Score Level	Description
Eyes	0	Best: eyes clear, clean, and bright.
	1	Moderate: Dirt and/or staining around the eye area. Any evidence of wet eye ring or inflamed eye lid.
	2	Worst: eyes sealed shut with or without conjunctivitis.
Nostrils	0	Best: nostrils with clean and clear air passageways.
	1	Dirty: nostril air passageways blocked with dust or mucus.
Feather Cleanliness	0	Best: clean and unstained breast, back feathers, or down depending on age.
	1	Dirty: adhering manure or staining on down or feathers.
Feather Quality	0	Best: good feather coverage for age—down in younger and feathers in developing and older birds
	1	Moderate: some evidence of feather picking; down and/or feather damage less than 2 cm^2^.
	2	Worst: Feathers/down damaged, short, and stubbly. Large patchy feathers/down over back greater than 2 cm^2^ and/or evidence of severe feather picking (presence of blood on back, tail, neck, or wings).
Foot Pad	0	Best: heel and toe pads free of any lesions or ingrained dirt.
	1	Moderate: Dirt pervades the heel or toe pads, and skin papillae raised, typically dark brown on heel or toe pads. Lesions covering less than 50% of heel or toe pad. Free of any bloody lesions.
	2	Worst: lesions or callouses cover 50% or more of heel or toe pad; any bleeding lesions.

^1^ This scoring system was described in our previous research [18].

## Data Availability

All data can be provided by the corresponding author upon request.
